# Shedding a new light on gastric cancer screening

**DOI:** 10.1002/ueg2.12600

**Published:** 2024-05-27

**Authors:** Anthea Pisani, Diogo Libânio, Lumír Kunovský

**Affiliations:** ^1^ Division of Gastroenterology Department of Medicine Mater Dei Hospital Msida Malta; ^2^ Department of Gastroenterology Porto Comprehensive Cancer Center Raquel Seruca, and RISE@CI‐IPO (Health Research Network) Porto Portugal; ^3^ MEDCIDS (Department of Community Medicine, Health Information, and Decision) Faculty of Medicine University of Porto Porto Portugal; ^4^ 2nd Department of Internal Medicine – Gastroenterology and Geriatrics, University Hospital Olomouc, Faculty of Medicine and Dentistry Palacky University Olomouc Olomouc Czech Republic; ^5^ Department of Surgery, University Hospital Brno, Faculty of Medicine Masaryk University Brno Czech Republic; ^6^ Department of Gastroenterology and Digestive Endoscopy Masaryk Memorial Cancer Institute Brno Czech Republic

**Keywords:** endoscopy, gastric cancer, screening

Early accurate detection of gastric cancer, which is the fourth most common malignancy worldwide,^1^ significantly improves morbidity and mortality. Whilst the 5 years relative survival risk of gastric cancer is 35.7%,^2^ when diagnosed at an early, resectable stage, 5‐year survival can approach 99% in selected screened populations.^3^

In view of this, several nations with a high prevalence of the disease have established screening programmes for gastric cancer in order to improve survival. Screening for early gastric cancer and its premalignant conditions such as intestinal metaplasia and mucosal atrophy has the potential to decrease the risk of death by 47%,^4^ yet this has proven to have many challenges.

Oesophagogastroscopy with tissue biopsy is currently the modality of choice to perform a diagnostic assessment of the stomach. This, however, also has some limitations, with meta‐analyses showing that 9.4% of gastric cancers are missed.^5^ Conventional white light imaging (WLI) has been used as the standard for endoscopic examination of the stomach to identify suspicious lesions; yet it has been shown to have a sensitivity between 33% and 75% for early gastric cancer with a specificity between 57% and 93.8%.^6^ Considering these pitfalls, various technologies have been developed to improve accuracy and decrease the rates of missed lesions, including high‐definition white‐light endoscopy, dye‐chromoendoscopy, virtual chromoendoscopy/image enhanced endoscopy (IEE—such as narrow‐band imaging [NBI], blue laser imaging [BLI], linked colour imaging [LCI], i‐scan and optical enhancement [OE]) and IEE‐magnifying endoscopy. Current ESGE guidelines recommend the use of virtual chromoendoscopy, with or without the use of magnification, for diagnosis of precancerous conditions and early gastric lesions.^7^ However, while the benefit of IEE is now well established for the detection and characterisation of intestinal metaplasia when compared with WLI, the benefit in the detection of early neoplastic lesions is less clear.

In this issue of the United European Journal of Gastroenterology, An et al.^8^ present the first prospective, large‐scale study utilising a new image enhanced endoscopic technology in the detection of gastric cancer: OE mode‐2 (OE‐2). The authors report that the advantages of OE‐2 include the ability to increase the overall brightness of the image by using red light emission as well as emission at around 415 nm which emphasises blood vessels on the mucosal surface and around 540 nm which emphasised blood vessels in the submucosa.^9^

The study is a prospective, multi‐centre, randomised‐control trial conducted across 16 hospitals in China. Patients at high‐risk for gastric cancer were randomised for inspection either with WLI or OE‐2. The study protocol is rigorous and well‐planned, with a large sample size in both groups. The authors report that OE‐2 was superior to WLI in detecting early neoplastic lesions, with a detection rate of 5.1% versus 1.9% (*p* < 0.001). This advantage was particularly prominent in those with a background of chronic atrophic gastritis, lesions of morphologic type 0‐IIa or 0‐IIb, in lesions containing high‐grade intraepithelial neoplasia and in lesions less than 2 cm in size.

These results are in line with previous reports that show increased detection rates with NBI, magnifying NBI, BLI and LCI. Although the authors of this study report that the main advantage of OE‐2 is to increase brightness, we should note that new generation IEE technologies (BLI‐bright, NBI‐bright, LCI) also have a brighter image when compared with the first version of these technologies, and that the use of other new IEE technologies have been shown to also increase detection rates.^10^, ^11^, ^12^

Another important message of these study is that the benefit was greater in patients with a background of chronic atrophic gastritis. Although a technology that increases detection should probably be also used in low‐risk cases, these results reinforce the importance of performing a high‐quality endoscopy with virtual chromoendoscopy in the surveillance of patients with known precancerous conditions, as recommended in ESGE guidelines.^7^

And finally, we should not forget that besides the technology used, there are also other important and operator related aspects. A high‐quality endoscopy that can improve detection should have good mucosal visibility (possibly with the use of defoaming agents and/or mucolytics), adequate sedation, adequate inspection time, a systematic inspection routine and accurate photo‐documentation to avoid blind spots, and of course, eyes trained in the detection and characterisation of subtle lesions.^13^ Image enhanced endoscopy has been shown to improve detection rates and is recommended by several societies and guidelines,^7^, ^14^ however artificial intelligence,^15^ including use convolutional neural network, might also have a role to play in the future.

The main message of this study is that we have another IEE technology available to improve detection rates. Although further confirmatory studies (namely in Western countries) are needed on the role of OE‐2, it has the potential to improve gastric cancer screening. Nevertheless, as discussed in this editorial, it is unlikely that one method will be the sole answer to enhance screening and a combination of the metrics of quality endoscopy is the key.

## CONFLICT OF INTEREST STATEMENT

The authors have no conflicts of interest to declare.

## Data Availability

Data sharing is not applicable to this article as no new data were created or analysed in this study.
